# Mobile Integrated Healthcare: Preliminary Experience and Impact Analysis with a Medicare Advantage Population

**DOI:** 10.36469/9819

**Published:** 2016-09-26

**Authors:** Daniel J. Castillo, J. Brent Myers, Jonathan Mocko, Eric H. Beck

**Affiliations:** 1 Evolution Health, Dallas, TX, USA; 2 Envision Healthcare, Greenwood Village, CO, USA

**Keywords:** mobile integrated healthcare, value-based care, interprofessional, community paramedic, care management, population health

## Abstract

**Background:** Mobile Integrated Healthcare (MIH) is a novel, patient-centered approach to population management. This concept creates a needs-matched, time appropriate assignment of one or more members of a multi-professional clinical team to care for patients on a scheduled or unscheduled basis. The selection of the site of care for scheduled interventions is driven by patient choice and, most often occurs in the patient’s home; unscheduled interventions are guided by a 5-point triage system and, based on acuity, may be treated in the home, primary care office, urgent care or, rarely, in an emergency department.

**Methods:** An MIH team was assigned to deliver a care coordination program for a Medicare Advantage PPO (MAPPO) population (55% female, 71.2 years mean age), with risk assignment and interventions designed to affect potentially avoidable utilization of Emergency Medical Services (EMS), emergency department, and medical inpatient admissions. Patients participating in the MIH program were compared with contemporaneous, risk-matched non-participants as well as to actuarially expected cost and utilization based on historical claim experience.

**Results:** All measured trends demonstrated favorable results for patients participating in the MIH program when compared against a matched cohort: 19% decrease in emergency department per member per month (PMPM) cost, 21% decrease in emergency department utilization, 37% decrease in inpatient PMPM cost, 40% decrease inpatient utilization, all measures reached statistical significance. Member experience satisfaction scores and patient activation measures also showed favorable preliminary trends.

**Conclusion:** This initial impact analysis of a MIH care coordination program for this MAPPO population demonstrates promising trends regarding utilization, cost, member experience and patient activation. These preliminary findings indicate both that implementation of such a program is feasible and strongly suggest meritorious impacts upon the health, experience and cost of care for the population.

## BACKGROUND

Improving health outcomes and the experience of care while simultaneously lowering healthcare costs continues to be a keen focus for healthcare providers, payers and other stakeholders in population health management.[1] Numerous factors contribute to escalating healthcare costs at the population level including: 1) fragmented systems of care, 2) challenges to access, 3) variable quality and 4) dissatisfying experiences.Provider sponsored care coordination interventions intuitively offer a rational approach to reducing healthcare spending and improving effective management of clinical conditions through patient-centered delivery. Considerable work in the fields of medical, clinical and care management, value-based healthcare policy, managed care, health economics, outcomes research and population health offer evidence supporting various interventions that can improve quality and health outcomes. There are, however, limited published and peer-reviewed examples of care coordination intervention that demonstrate an increase in quality and reduction in costs – or more specifically, improved health and experience outcomes with concurrent financial savings.[2,3,4,5,6]

Complexity of the US healthcare system, persistent inefficiencies and discontinuities in pursuit of health are perhaps most pronounced in the care of the chronically ill, frail and high-risk aging populations at home. A perpetually rising cost trend, high utilization of 911, Emergency Medical Service (EMS) transport, emergency department (ED) and inpatient services, frequent hospital readmissions, and the inevitable complication and frustration that results from such patterns may be significantly prevented or avoided.[7] There are numerous single-purpose healthcare providers and services offering important, but niche, clinically-narrow care, often only under specific circumstances (ie home-bound status, end of life, perceived emergency) and limited availability during 'non-business hours.' Mobile Integrated Healthcare (MIH) is a novel emerging delivery model designed to address these problems in a scalable manner. At its essence, MIH is needs-based, patient-centered, 24/7 acute care, chronic care and prevention services delivered in the home or mobile environment by the cost-effective synchronization of various clinicians, infrastructure and resources.[8] It is a framework for an intentional strategy used to improve health, quality, integration and cost of care for a defined population or subpopulation.

Inspired and informed by outcome improvement and experiences previously reported using pre-hospital or out-of-hospital systems of care to serve subpopulations with defined needs, the MIH model mirrors program planning and evaluation methods used by community, public and population health practitioners.[9] Examples include trauma, stroke, cardiac arrest and heart attack systems of care that link community prevention efforts, 911 communications, EMS clinicians, hospitals and specialists in an organized approach to delivering evidenced based care that can improve morbidity and mortality.[10] Similarly, using the complimentary clinical expertise, professional competencies and various existing delivery models including home health, home hospice, transitional care, telephonic coaching, community paramedicine, home based primary care, mobile clinic, telemedicine and telephonic triage, MIH interventions capitalize on these traditionally unsynchronized resources through a more intentional and unifying system approach to improve outcomes.[11] Such delivery requires enabling revenue models for intervention, logistics and workflow technology to link disconnected services, processes that support information exchange across multiple care settings and willing prepared team members for collaborative interprofessional team-based delivery.

Numerous examples highlight the potential value creation from various MIH intervention activities. Appreciating that acute care is common, accounting for over one third of all patient encounters, it is not surprising that approximately 8% of the US population access health care via 911/EMS each year.[7,12] Studies suggest that up to 34% of Medicare beneficiaries transported by EMS could have been treated safely in an alternate setting and at least 80% of unscheduled hospital admissions resulted from an ED visit.[12,13,14,15] Alpert et al reported approximately $600 million in annual savings could safely be achieved if Medicare beneficiaries with low-acuity needs were transported by EMS to settings other than an emergency department. If the same patient-centered EMS transport redesign was enabled by Centers for Medicare and Medicaid Services (CMS) and third-party payers, estimated annual savings could reach $1.2 billion.[16]

The Wake County EMS System in North Carolina has published MIH experience on safe alternative triage to avoid unnecessary transport of assisted-living facility residents who have unintentional falls, a costly and undesirable scenario occurring some 2.4 million times per year in the United States. In this example, to safely avoid transport the EMS physician medical director instituted a validated protocol and partnered with visiting geriatric physician services to coordinate care and limited unnecessary transport.[17] Additionally, there are numerous subpopulation-specific programs and CMS demonstrations in which providers have partnered with behavioral health professionals, EMS, home hospice nurses, care managers, primary care and hospital case managers respectively to drive improved 24/7 care coordination and patient outcomes. Aside from financial and clinical outcome improvement, one MIH hospital readmission reduction program, inclusive of paramedics, advanced practice providers, nurses and physicians providing in-home and telephonic support has been reported to improve patient experience.[18] Common to these various MIH programs are multi-professional teams, integrated services and workflows across conventional practice settings and team composition to deliver subpopulation-specific intervention under physician oversight.

No studies to date have reported quantitatively or explicitly examined the MIH intervention model. This study aims to describe and analyze the initial experience and preliminary impact of an MIH intervention delivered at scale for a high-risk subpopulation. Using both actuarial and epidemiological frameworks, we reviewed available paid claims, administrative, operational and clinical data, to assess the association and potential relationship between the MIH intervention and its health, experience and financial impact.

## Study Setting

This retrospective observational study was conducted utilizing the experience and data from an MIH care coordination program for the state-wide membership of a Medicare Advantage Preferred Provider Organization (MAPPO) population in Florida. This MIH care coordination program was administered by Florida Outpatient Services, P.A. (FLOS), an integrated mobile medical practice, managed by Evolution Health, specialized in the care of complex and vulnerable patients in the home and alternative settings. The MAPPO membership enrollment at the start of the MIH intervention in November of 2015 was 61,804 with 55% being female with a mean age of 71.2. The average hierarchical condition categories (HCC) risk score for the population was 1.07 at that time and increased to 1.15 at the beginning of 2016 due to the progression of disease in carry-over members and new enrollees' increased risk. The members were geographically distributed across the state, but with higher membership density in counties with larger metropolitan cities, namely Miami, Fort. Lauderdale and Tampa.

The physician-led MIH care team consisted of multiple clinical professionals including emergency medical technician (EMT), paramedic, nursing, social worker, pharmacy and advanced practice provider personnel. Outreach mechanisms including direct mail, telephone calls, emails and in-hospital bedside pre-discharge visits. These touch points were used to engage individual members of a select target population. Members who consented to participate were enrolled in the MIH intervention and prospectively risk stratified using the Patient Activation Measure (PAM) to guide intensity of MIH program intervention activity. The PAM is a standardized 13-item tool most often used to quantify a patient's engagement, activation, or self-management capabilities.[19] Of note, PAM is not condition specific; rather it is designed to assess a person's knowledge, skill, confidence and readiness related to managing and advocating for his or her health and associated care. Significant evidence supports the link between the patient activation measure and health outcomes, patient experience and costs.[20] PAM can be used to guide interventions for maximal return, as an intervention program process measure, as a leading indicator predictive of cost, utilization and experience, or as a population-level impact outcome.[21,22]

The MIH program interventions included evidenced-based, interprofessional, clinician-delivered population health and care management activities targeting impactable or intervenable high risk members based on potentially avoidable cost. Specifically, this MIH intervention focused on supporting members' needs and coordinating care during transitions from one care setting to another (ie transitions of care [TOC] from hospital to home, hospital to nursing facility, nursing facility to home and/or ED to home), longitudinal management for high risk (LHR) and chronically ill members and palliative support for members with advanced chronic illness (AIM). All enrolled members had a care plan created by a nurse practitioner or physician assistant to guide team-based care coordination and intervention activity in collaboration with the member's primary care and/or specialist physicians. All engaged members had access to 24-hour-a-day services including a telephonic hotline for on-demand unplanned care needs. These unscheduled in-bound calls, or unplanned care (UPC) calls, were triaged by nurses and resulted in time-appropriate call navigation based on need. Unplanned care call dispositions included on-demand in-home clinician visits, telephonic consultation with prescribing provider, social worker or pharmacist, telemedicine encounters, scheduled follow up with MIH clinician or in-network provider or other non-clinical support services (eg transportation, advocacy, community resources).

Based on a member's PAM and clinical intervention program (TOC, LHR, AIM) an assigned standard schedule for planned activities – weekly phone calls and in-home encounters – was established with 24/7 access to unplanned care services available to all engaged members on-demand. Tailored coaching based on PAM, diagnosis, clinical conditions, medication and care plan adherence, coordination needs and changes in member status guided the scope and content for planned scheduled encounters. Self-management coaching, outpatient appointment follow up, medication teaching, diagnosis-related education and reinforcement of available alternatives to 911, EMS, the emergency department and hospital that would allow a member to remain at home were key intervention encounter content themes.

We defined an engaged member as any member who the MIH care team spoke to, either in-person or telephonically, and explained the clinical model, offered enrollment, and provided the UPC telephone number. We defined an enrolled member as any member who was engaged and subsequently consented to one of the MIH clinical intervention programs (LHR, AIM, TOC).

## STUDY DATA AND METHODS

### Data

While this care coordination program is ongoing, we describe and analyze the initial tranche of members targeted for intervention. The principle data sources for this study were the enrollment and medical claims files for the state-wide MAPPO membership of a large payer from May, 2015 through April, 2016. This time period was selected because it allowed the identification and sufficient measurement of the membership's per member per month (PMPM) incurred and paid claim costs as well as the corresponding utilization trend for 6 months prior to the program start date of November 1st, 2015. This interim analysis and reporting period allows for 3 months of program participation with an additional 3 months of claims run-out. The program was designed to lower costs and utilization specifically for preventable emergency department and inpatient utilization, so the corresponding claim cost categories (Milliman Healthcare Cost Guidelines grouper logic) were used to consistently group claims and support impact measurement.

Program outreach, engagement and enrollment information was obtained from the FLOS internal logistics operating and scheduling platform. Members that enrolled and participated in the MIH program were scheduled and tracked using this logistics platform, enabling the accurate and timely monitoring of all intervention activities and the respective task times associated with each.

Targeting interventions to subpopulations with certain high-risk features has shown greater benefit compared to intervention across the broader general population.[23,24,25] The Milliman PRM Analytics tool was licensed for identification and prediction of impactable actuarial and intervenable clinical risk, by estimating potentially avoidable cost (PAC).[26] Two years of pre-program historical paid claims data for the entire MAPPO population were analyzed. We determined each members' baseline characteristics, utilization and PMPM costs from the health plan's enrollment and claims data filtered to include only ED and inpatient medical spend. These target members were then placed into separate risk cohorts (levels 1, 2 and 3 respectively) by ordinally ranked PAC – with level 1 being those members with the highest PAC as predicted over the next 6 months. The experience and analysis in this study reflects the initial tranche of members, risk level 1, specifically targeted for the MIH program. Figure 1 summarizes this target member identification, outreach and enrollment process.

**Figure 1.** Member Classification

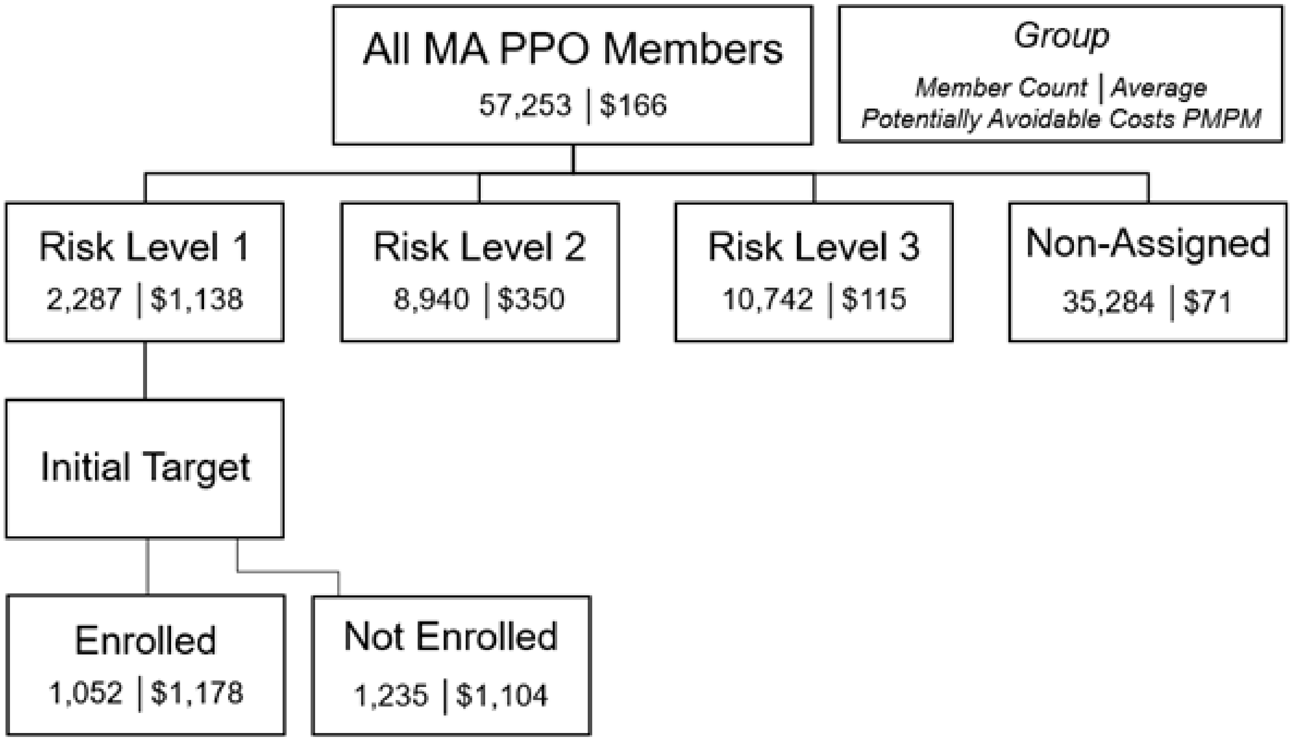

Impactable subpopulations identified as intervention targets based on historical claims data and predicted potentially avoidable cost.

The PAM[27] was collected prospectively from enrolled members during the program's initial encounter and serially every 60 days or with change in patient status for the duration of the program. Patient experience data was collected anonymously via a third-party service.[28] Data on the experience of care included overall program satisfaction and multiple additional dimensions such as likeliness to recommend program, ease of scheduling, perceived clinician competence and empathy. Patient experience information was collected after the initial visit, and serially every 60 days for the duration of program participation.

## Analysis of Data

The intervention cohort was comprised of targeted members who enrolled and received the MIH coordination intervention; the control group was comprised of targeted members who did not enroll and did not receive the intervention. The intervention and control groups were matched by Milliman PRM Analytics tool PAC calculation and standardized by CMS Hierarchical Condition Category (HCC) risk scores. HCC risk scores were allocated by member and accumulated monthly based on member eligibility. The average of the monthly accumulated HCC risk scores were used to level the cost or utilization metrics for a given month, where the cost and utilization metric is the numerator and the average monthly HCC risk score is the denominator. This HCC risk adjustment application normalizes both cohorts for two different types of metrics, cost and utilization. The Milliman PRM Analytics tool PAC is based on the managed care standard and published retrospective claims research from the Agency for Healthcare Research and Quality (AHRQ) Ambulatory Care Sensitive Conditions and the New York University Center for Health and Public Service Research Emergency Department Utilization algorithms.

The NYU algorithm identifies the following potentially avoidable ER classifications: non-emergent (immediate medical care was not needed within 12 hours, eg sore throat); emergent/primary care-treatable (treatment was needed within 12 hours, but care could have been provided effectively and safely in a primary care setting, eg ear infections); and emergent/ER care needed but preventable/avoidable (ER care was needed, but patients may have been able to avoid the emergency medical issue if they had received time and effective outpatient care while they were sick, eg exacerbation of chronic diseases such as asthma, diabetes or congestive heart failure).[29,30,31] Ambulatory care sensitive conditions (ACSCs), are conditions for which good outpatient care can potentially prevent the need for hospitalization, or for which early intervention can prevent complications or more severe disease.[32] For each member in the population, the Milliman PRM also predicted an estimation of potentially avoidable healthcare expenses and utilization in the ensuing six-month period, absent additional management or intervention. This MIH program elected to focus on retrospectively identified PAC, members having prospective characteristics with the largest risk for PAC and the opportunity for impact from intervention. The same analysis and targeting was applied to both the intervention group and the control group, and this equivalence is demonstrated in tables 1 and 2.

Enrolled members in the intervention cohort were those that consented to the program and received a scheduled welcome visit. The start date was defined as the date of their welcome visit – the first in-person interaction with an MIH clinician. The member's cost, utilization and trend for inpatient medical facility and professional, and emergency department facility and professional, were calculated for both 6 months prior to the welcome visit and 3 months after. In this process, 3 months of claims run out was held constant in assessing cost, utilization and trend to maintain uniformity in claim completion for measurement periods. Each member in the intervention cohort remained in the program for the full 90 days to ensure uniform exposure to the intervention. Those members who did not have a full 90 days of intervention experience were excluded from the intervention cohort, as this was rare and only occurred when the member lost MAPPO eligibility. The control cohort was established from those members who were targeted for engagement and enrollment, but did not complete a "welcome visit" and thus were never categorized as enrolled. These members were assigned a start date of January 1st, 2016 as this provided 6 months of pre-program data to be analyzed as well as 3 months of program data and 3 months of claims run out. Using this date also minimized potential impact from seasonality as the mean welcome visit of the enrolled members was January 1, 2016.

All intervention activity was tabulated by member and by cohort, this included the outreach and engagement to targeted members, planned intervention encounters as well as unplanned intervention encounters (Figure 2).

**Statistical Analysis**

A paired t-test was conducted on both cohort's HCC scores to evaluate differences in pre-program risk levels (Table 1). This was followed by a multivariate analysis of variance (MANOVA) test of each cohort's 7 health status variables listed in Table 2. The results in tables 1 and 2 demonstrate that the characteristics of the intervention and control groups have reasonable overlap and are not statistically different.

To analyze and compare effectiveness, given the measurement focused on a group of high-risk patients during a 9-month period – a 6-month period pre-program, and 3-month period after joining the program - we based our evaluation on each member's cost record and a utilization record for each month, and for each service type. As the records were for a matched population, we had two sets of paired samples, one set is the means of cost/count of visits during the pre-program period, and the other is the set of means of cost/count of visits during the post-program period. Paired student t-tests were conducted to compare the samples.

**Table 1.** Baseline Characteristics of Intervention and Reference Population used as Control Group

| | Intervention (n = 1,074) | Control (n = 1,241) | P-value |
|---|---|---|---|
| **Demographics** | | | |
| Age, mean | 72.56 | 73.19 | 0.2408 |
| Female patients | 57.9% | 57.1% | 0.4534 |
| HCC Risk Score, mean | 3.04 | 3.19 | 0.25 |
| PAC (6 months) | $6588 | $6649 | 0.7255 |
| **Health Status** | | | |
| CHF | 23.0% | 28.8% | 0.0032 |
| COPD | 37.8% | 40.7% | 0.2711 |
| Diabetes | 46.0% | 49.6% | 0.1696 |
| Dementia | 12.2% | 13.8% | 0.3417 |
| CKD | 30.8% | 34.8% | 0.0689 |
| CAD | 42.4% | 47.0% | 0.0572 |
| CVA/TIA | 20.2% | 22.8% | 0.1770 |
| MANOVA test | | | 0.1097 |

CAD: coronary artery disease; CHF: congestive heart failure; CKD: chronic kidney disease; COPD: chronic obstructive pulmonary disease; CVA: cerebrovascular accident; HCC: Hierarchical Condition Category; MANOVA: multivariate analysis of variance; PAC: potentially avoidable cost; TIA: transient ischemic attack

**Table 2a.** Unadjusted PMPM Costs, Utilization and Trends

| | Pre | Post | | Pre | Post | | |
|---|---|---|---|---|---|---|---|
| | **Intervention (n= 1,074)** | | | **Control (n = 1,241)** | | | **P-value** |
| PMPM (3 mo. mean) | | | | | | | |
| Total | $934.60 | $961.31 | | $862.72 | $925.01 | | 0.00931 |
| Inpatient | $509.61 | $520.71 | | $397.45 | $426.78 | | 0.00000 |
| Emergency Room | $84.84 | $74.59 | | $72.42 | $79.57 | | 0.00000 |
| Utilization (3 mo. mean) | | | | | | | |
| Inpatient (per 1000) | 76.53 | 69.30 | | 63.39 | 75.60 | | 0.00000 |
| Emergency Room (per 1000) | 138.01 | 134.54 | | 129.20 | 154.16 | | 0.00000 |
| PMPM Trend (6 mo. pre, 3 mo. post) | | | **Difference** | | | **Difference** | |
| Total | 14% | -19% | -33% | 10% | 5% | -5% | |
| Inpatient | 19% | -21% | -40% | 13% | 8% | -5% | |
| Emergency Room | 17% | -6% | -23% | 5% | 8% | 3% | |
| Utilization Trend (6 mo. pre, 3 mo. post) | | | **Difference** | | | **Difference** | |
| Inpatient | 28% | -18% | -46% | 6% | 6% | 0% | |
| Emergency Room | 19% | -5% | -24% | 4% | 7% | 3% | |

**Table 2b.** Risk-adjusted PMPM Costs, Utilization and Trends

| | Pre | Post | | Pre | Post | | |
|---|---|---|---|---|---|---|---|
| | **Intervention (n= 1,074)** | | | **Control (n = 1,241)** | | | **P-value** |
| PMPM (3 mo. mean) | | | | | | | |
| Total | $314.54 | $318.68 | | $270.83 | $346.15 | | 0.00931 |
| Inpatient | $159.58 | $172.60 | | $124.77 | $161.11 | | 0.00000 |
| Emergency Room | $26.77 | $24.73 | | $22.73 | $30.78 | | 0.00000 |
| Utilization (3 mo. mean) | | | | | | | |
| Inpatient (per 1000) | 25.76 | 22.97 | 19.90 | 23.76 | | | 0.00000 |
| Emergency Room (per 1000) | 46.73 | 44.61 | 40.56 | 48.45 | | | 0.00000 |
| PMPM Trend (6 mo. pre , 3 mo. post) | | | **Difference** | | | **Difference** | |
| Total | 11% | -19% | -30% | 10% | 5% | -5% | |
| Inpatient | 16% | -21% | -37% | 13% | 8% | -5% | |
| Emergency Room | 14% | -5% | -19% | 5% | 8% | 3% | |
| Utilization Trend (6 mo. pre , 3 mo. post) | | | **Difference** | | | **Difference** | |
| Inpatient | 23% | -17% | -40% | 6% | 6% | 0% | |
| Emergency Room | 16% | -5% | -21% | 4% | 7% | 3% | |

PMPM: per member per month

## RESULTS

During the 3-month intervention period, 1074 members were enrolled in the program, and 1241 members were targeted but not enrolled. At baseline, the enrolled and non-enrolled populations were similar in age, gender, number of chronic conditions, risk scores, and PACs (Table 1).

The pre-program PMPM costs, utilization and trends were calculated for both the intervention and control cohorts. Table 2 shows both the unadjusted and risk-adjusted PMPM costs, utilization, and trend of ED and inpatient medical services for both cohorts for the 6 months prior to the start of the program. Since both cohorts were targeted for intervention using the same PAC calculation, and that both cohorts had similar baseline PMPM costs and utilization for both ED and inpatient we find them equivalent, appropriate for trend comparison and impact analysis.

### Utilization, Costs and Estimated Savings

Preliminary results of the risk-adjusted analysis of utilization and cost trends conducted by Evolution Health's Population Intelligence team showed a 37% decrease in the enrolled group's PMPM cost trend for inpatient medical and a 19% decrease in PMPM cost trend for emergency department as compared to the non-enrolled group's 5% decrease in PMPM cost trend for inpatient medical and 3% increase in PMPM cost trend for emergency department. The utilization trend analysis showed a 40% decrease in inpatient medical utilization trend for the enrolled group and a 21% decrease in the ED utilization trend. This compares to the non-enrolled group's 0% change in inpatient medical utilization trend and 3% increase in ED use. Each of these differences in trend demonstrated statistical significance, with the intervention group having significantly lower trends in PMPM costs and utilization for both inpatient medical and emergency department categories (Table 2). Also of interest, is that the PMPM trend inclusive of all cost categories (not just inpatient medical and ED) was also significantly lower for the intervention group – implying that the impact of cost reduction in inpatient medical and ED cost categories can lead to significantly lower costs overall. This is not surprising as inpatient medical and ED cost categories often comprise a large portion of the total cost of care, and also a large share of cost that can be potentially avoided.

To calculate the expected savings generated from the program, we used the Actuarial-adjusted Historical Control Methodology.[33] We first applied the trend of the control group post-intervention to the baseline PMPM from the 6-month pre-program analysis of the intervention group. This trend was used to determine the expected PMPM costs for the intervention cohort absent the MIH intervention. $287 000 in savings was calculated (Table 3) by netting the actual PMPM costs for the intervention group from this expected PMPM cost calculation.

### Activation and Experience

The initial PAM was obtained from enrolled members during their initial visit. This was recorded as their baseline PAM. This measure was then used to tailor the MIH model for improved activation through higher touch, coaching for activation and increased engagement for those with a lower PAM score. The initial PAM for enrolled members had a mean score of 65.09. This score has improved to 68.51 over 3 program months (Table 4). This 5.3% increase may correlate to an expected decrease in hospital utilization of 6.8% based on previous peer-reviewed publications on the predictive use of PAM.[21,22]

In addition, member satisfaction was high, as anonymously measured by a third-party compilation and analysis of member surveys over the course of the intervention period. As summarized in Table 5, 97% of the respondents agreed or strongly agreed that the providers communicated clearly. Ninety-seven percent agreed or strongly agreed that their provider was knowledgeable, and 96% indicated they would recommend the services to their friends and family.

**Table 3.** Estimated Savings

| | | +30 Days | +60 Days | +90 Days | Total |
|---|---|---|---|---|---|
| [Intervention Group Baseline PMPM @ -30] x [5% Control Group Post Cost Trend] | | $371.45 x 1.05 = $390.02 | $409.52 | $430.00 | |
| Minus: | Actual Intervention Group Cost PMPM | $379.54 | $324.87 | $250.12 | |
| Equals: | Estimated Savings PMPM | $10.46 | $84.66 | $179.88 | |
| | | | | | |
| Multiplied by: | Membership | 1069 | 1043 | 1043 | |
| | | | | | |
| Monthly Estimated Savings | | $11 000 | $88 000 | $188 000 | $287 000 |

PMPM: per member per month

**Table 4.** Intervention Group Change in Patient Activation Measures

| | |
|---|---|
| Mean initial PAM Score | 65.09 |
| Latest PAM Score | 68.51 |
| Change | 3.42 |
| % Change | 5.3% |
| 284 patients with 2 or more PAM scores assessed | |

PAM: patient activation measures

**Table 5.** Intervention Group Patient Experience Results

| | Agree | Strongly Agree | Total | Statements |
|---|---|---|---|---|
| **Communication** | 22.30% | 74.30% | 96.6% | My provider actively sought my opinion; my provider carefully listened; my provider clearly communicated my options |
| **Knowledge** | 15.95% | 81.52% | 97.4% | My provider was knowledgeable |
| **Net Promoter Score** | 23.20% | 72.16% | 95.8% | I would recommend my provider to family and friends |
| 396 surveys between Feb 16 (survey start) and July 31 | | | | |

## DISCUSSION

We describe the initial observations and impact of a MIH program for a MAPPO population. While it has been hypothesized that MIH programs can improve outcomes, improve the patient experience, and reduce costs at the population level, this is one of the few (if not the only) analyses that indicates the potential magnitude of actual impact.

We have utilized analytical methods that incorporate clinical, epidemiologic, fiscal, and actuarial expertise. Thus, the initial analysis of the population, risk stratification, targeted interventions, and analysis of impact represent a uniquely robust view of the population. Specifically, we compared the population's utilization based on well-established historical actuarial analysis while incorporating epidemiologic principles to compare intervention and control groups in a contemporaneous manner. The difference in cost and utilization described in the results are corroborated by the well-established evidence-base for the component interventions delivered as part of this MIH program. These evidence-based interventions include timely support during transitions, medication review, scheduled interventions for those at high risk for clinical deterioration, appropriate referral to palliative care, and 24/7 unplanned care access.

Demonstrating intervention impact convincingly has proven to be difficult when the primary outcome of interest is the financial savings, or something that did not occur. Given that most population health and care management interventions are not evaluated based on double-blind randomized control trials, it is worthwhile to utilize established methods from epidemiology, health services, observational and outcomes research to ensure measurement validity. Classic epidemiologic studies of disease etiology examine the possible relationship between a putative cause, the independent variable, and an adverse health effect or effects, the dependent variable. In doing so, such studies take into account other factors, including health care, that may influence this relationship or confound it. Health services and outcomes research focuses on health services or program interventions as the independent variable, with a reduction in adverse health effects as the anticipated result, the dependent variable if the care or intervention is effective. In this case, environmental and other factors that may modify the relationship are also taken into account. The 1964 US Surgeon General's report Smoking and Health establishing the link between smoking and lung cancer, as well as subsequent work by Sir Austin Bradford Hill and others have defined well-recognized criteria for causality. These criteria are important considerations and may be applied when assessing whether a particular exposure or intervention is causally associated with certain given health outcomes in an observational setting.[34,35] The basic elements required in considering potential causal inference include: strength of association, temporality, consistency, theoretical plausibility, specificity, dose response relationship, experimental evidence, coherence, and analogy.[36,37]

Wilson and MacDowell[38] refer to a "causal pathway" nomenclature and define supporting Type I, Type II and Type III metrics for evaluating intervention causality in health program analysis. "Type I" metrics determine the basic components involved in the program or intervention processes and are typically inputs rather than process results. For example, number of members enrolled in the intervention program. "Type II" metric is a process outcome, intermediate measure that represents a proximate result to the ultimate outcome. For example, number of unplanned care intervention encounters utilized. "Type III" metrics are the specific target outcomes for which the program or intervention was designed – clinical, quality, economic, satisfaction, etc. For example, per member per month medical claims expense. These three types of metrics can be used to measure the program intervention and correlational association in assessing a potential causal relationship between intervention exposure and outcomes.[33,37] Figure 2 summarizes the causal pathway framework for this MIH intervention program.

**Figure 2.** Causal Pathway Metric Types and Corresponding Data

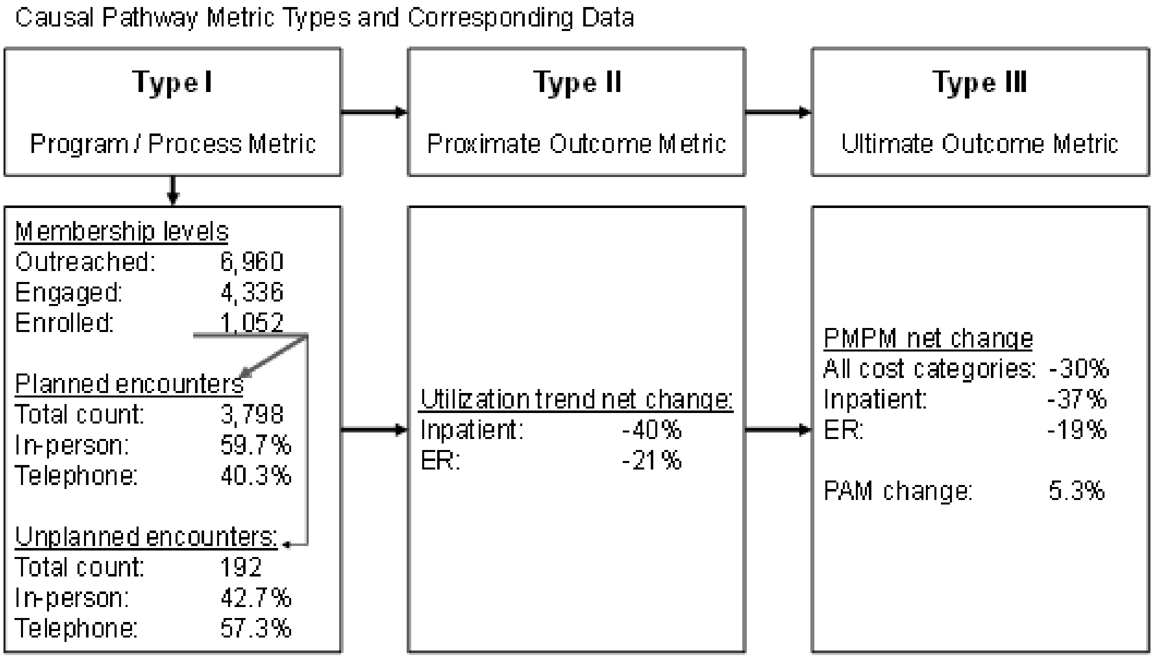

PAM: patient activation measure; PMPM: per member per month

Our interpretation of the data and results presented here are consistent with the framework outlined. Previous literature has established the evidence base underpinning the content of the MIH intervention component activities, specifically, transitions of care support, PAM coaching for activation, advanced illness and high-risk member care coordination. Given the statistical significance of this interim analysis of utilization and cost, and that the notable change in trend is isolated to the intervention cohort, following the institution of the intervention program, we find good support for causal inference. Namely, there is strength in the association, and temporally the intervention precedes the effect. The intervention and outcomes are theoretically plausible based on previously reported evidence and the impact is isolated to the intervention cohort in a manner that is supported by the underlying intervention activity and process metrics. Additional MIH program experience, the passage of time to allow claim development and sustained program impact, as well as program maturity and a larger enrolled membership in the intervention cohort (including those from the risk level 2 and 3 targeted subpopulations) will inform more robust analysis around the consistency, specificity, dose response and coherence dimensions of causal inference and program effectiveness.

A single study will not provide sufficient evidence to definitively establish the value of MIH or related interventions. To this end, our initial experience, description and preliminary impact analysis provides a foundational review with the subsequent work to include review of the mature program, larger intervention experience and additional passage of time to allow medical claims run out and completion. The study results indicate a clear directional change and impact on the intervention cohort, we look forward to meaningful quantification that can be reported in the future, after interval development occurs.

## Limitations

Given that members self-selected to enroll in the program, surveillance bias may have been introduced. However, both the intervention and control cohorts were identified using the same targeting algorithm and PAC values, as well as the other dimensions of comparison including age, gender, chronic conditions – all of which were similar (Table 1).

Regression to the mean may be an additional concern due to the high risk of the members targeted for intervention. It is important to note that regression to the mean is more of an individual phenomenon than population level observation. Any regression to mean should be approximately equal in both groups given that the reference population used as a control in this study was derived using the same objective criteria and that equivalence has been demonstrated between the cohorts.

Impact and outcomes measurement must consider the role of correlational association, causal inference and the various standards of support requisite in actuarial, financial, epidemiological and savings analysis. Evaluating and establishing causality in health intervention programs requires that the mechanism whereby outcomes achieved are unambiguously demonstrated and traceable to the specific intervention(s).[33] The preliminary and interim nature of this analysis only allows partial assessment of correlation association and causal inference at this time, and remains a descriptive report of the program experience to date.

Differences between our actuarially expected projections and the actual incurred and paid amounts for all studied groups depend on the extent to which future experience conforms to the assumptions made for this analysis. It is certain that actual experience will not conform exactly to the assumptions used in this analysis.

In performing this analysis, we relied on data and information provided by the MAPPO payer, consistent with industry standards and regulatory requirements. We performed a limited review of the data integrity used directly in our analysis for reasonableness and consistency and have not found material defects in the data. The exact claim liabilities will only be determinable after a significant passage of time.

The preliminary nature of this analysis and the ongoing status of this program limit sufficient visibility to meaningfully calculate intervention resource and infrastructure cost, and report a more detailed description of specific intervention components at this time. Resource utilization of marginal capacity, start-up costs and task time analysis are critically important in accurately reflecting operating expenses associated with delivering the MIH intervention. Full review of the TOC, LHR, AIM programmatic components, resource and infrastructure costs, as well as comprehensive analysis of the return on investment for intervention will be important in the follow up reporting on the mature MIH program experience.

## CONCLUSIONS

This report of initial experience and intervention impact of a MIH care coordination program for a Medicare Advantage population demonstrates promising trends regarding the potential value of such work. A framework for the descriptive analysis, program effectiveness and causal association has been offered for an MIH program designed primarily to reduce avoidable utilization and cost, and secondarily improve patient experience and activation. While we anxiously await more complete data from additional claim experience and anticipate richer analysis after the passage of additional time, program maturity and larger cohort of enrolled members, these preliminary findings indicate both that implementation of such a program is feasible and strongly suggest meritorious impacts upon the health, experience and cost of care for the population.

## ACKNOWLEDGEMENTS

Paul Hinchey, MD, MBA; Jeffrey Beeson, DO, RN, EMT-P; Sean Cannone, DO, CMD; Jonathan Sague, MSN, ACNP-BC, EMT-P; Brandy Sparkman, RN, BSN; Samuel Brown, MBA, EMT-P; Scott Bourn, PhD, RN, EMT-P; Greg Scott, RN, EMT-P; Sean Burton, EMT-P; James Musiyo, MCIS; Gayle Ballard; William Sanger; Randall Owen; Ted Van Horne; Todd Zimmerman; Ed Racht, MD; Dighton Packard, MD; Melissa McNally, MHSc, PA-C, EMT-P; Alex Moore, RN; Brendan Anzalone, DO; Joseph Nelson, DO; Bill Hall; Tom McEntee; Daniel Connor; Jiaquan Zhou; Ajay Gupta; Herschel Sharp; Sharon Henry, John Myers, MD; Steve Murphy, RN; Evolution Health, AMR, EmCare and the Envision Healthcare Corporation.
